# Effect of Exchange-Correlation Functionals on Schottky Barriers at Si/Metal Interfaces

**DOI:** 10.1021/acs.jpcc.6c01668

**Published:** 2026-06-03

**Authors:** Viviana Dovale-Farelo, Kamal Choudhary

**Affiliations:** † 10833 National Institute of Standards and Technology, Gaithersburg, Maryland 20899, United States; ‡ University of Maryland, College Park, Maryland 20742, United States; § Department of Materials Science and Engineering, Johns Hopkins University, Baltimore, Maryland 21218, United States; ∥ Department of Electrical and Computer Engineering, Johns Hopkins University, Baltimore, Maryland 21218, United States

## Abstract

Accurate prediction of Schottky barrier heights (SBHs) at metal–semiconductor interfaces is essential for understanding and optimizing charge injection in electronic and optoelectronic devices. However, first-principles calculations of SBHs remain challenging due to semiconductor bandgap underestimation, metal Fermi level placement, lattice mismatch, geometric alignment, and electrostatic potential alignment across the interfaces. In this work, we present a systematic and physically grounded assessment of computational strategies for SBH prediction using Si(111)/metal (Al, Cu, Ag, Au) interfaces as representative test cases. We evaluate multiple exchange-correlation treatments in combination with three distinct bulk reference protocols: relaxed, relaxed with spin–orbit coupling, and strained, consistent with the interface geometry. Benchmarking against experiment demonstrates that structural and electrostatic consistency between interface and bulk reference calculations is the dominant factor governing SBH accuracy. Mixed hybrid-semilocal approaches combined with strained reference protocols yield uniformly positive and significantly improved SBHs, achieving near-experimental accuracy with favorable computational cost and predictive performance.

## Graphical abstract

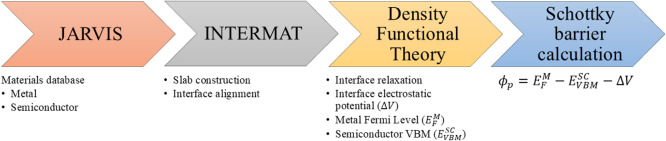

## 1 Introduction

Metal–semiconductor (M-SC) interfaces play a central role in determining the performance of electronic and optoelectronic devices, including diodes, transistors, and sensors.^1,2^ A key property governing charge transport across these interfaces is the Schottky barrier height (SBH), which controls carrier injection from the metal into the semiconductor. The SBH originates from the alignment of the metal Fermi level (E_*F*_) with the electronic band structure of the semiconductor upon contact.^3,4^ For electrons, the barrier height Φ_n_ is defined with respect to the conduction band minimum (CBM), while for holes, the barrier height Φ_p_ is referenced to the valence band maximum (VBM).^5^ These barriers directly influence contact resistance, turn-on voltage, leakage current, and overall device efficiency.

Accurate knowledge of Schottky barriers is therefore essential for the rational design and optimization of electronic and optoelectronic components. In modern nanoscale devices, contact properties often dominate device performance, making reliable prediction of SBHs a prerequisite for materials selection and interface engineering. As device dimensions shrink and new material combinations are explored, predictive modeling of metal–semiconductor^6^ contacts becomes increasingly important.

Despite their conceptual simplicity, Schottky barriers remain challenging to predict quantitatively from first principles. Standard density functional theory (DFT)^7^ approaches are limited by the well-known bandgap underestimation problem associated with commonly used exchange-correlation (XC) functionals such as the local density approximation (LDA)^8–10^ and generalized gradient approximation (GGA).^11^ More advanced functionals, including meta-GGAs and hybrid functionals, improve the description of semiconductor bandgaps but at substantially higher computational cost, particularly for the large supercells required to model realistic interfaces.^12,13^ In addition, the accurate placement of the metal Fermi level, the treatment of interfacial dipoles, and the alignment of electrostatic potentials across the interface introduce further sources of uncertainty. As a result, different computational studies often report widely varying SBHs for the same material systems.

Beyond electronic structure limitations, constructing realistic atomistic models of M-SC interfaces is itself nontrivial. Lattice mismatch, surface terminations, and interfacial reconstructions can strongly influence the local bonding environment and electrostatic potential profile. Consequently, many existing studies focus on a small number of idealized interfaces or employ system-specific modeling choices, limiting the generality and transferability of their conclusions.^1^ Comprehensive and consistent assessments of Schottky barrier predictions across different materials and computational protocols remain relatively scarce.

Previous first-principles studies have established DFT as a powerful framework for investigating Schottky barriers at metal–semiconductor interfaces, while also highlighting persistent methodological challenges.

Delaney et al. provided a detailed DFT treatment of Schottky-barrier formation at epitaxial ErAs/GaAs interfaces, using macroscopic averaging of the electrostatic potential to align the metal Fermi level with the semiconductor band edges.^14^ Their results emphasize that the SBH is highly sensitive to interface orientation, structural details, strain-consistent reference calculations, and DFT bandgap underestimation.

Stengel et al. extended this framework to metal/ferroelectric interfaces and showed that DFT bandgap errors can lead to pathological band alignment, in which the apparent Schottky barrier becomes negative and causes unphysical charge spill-out into the insulator, ultimately making the predicted Schottky barriers unreliable.^15^

Later, Wang et al. studied thermionic transport in layered van der Waals heterostructures.^16,17^ Their work shows that predicted barrier heights and currents are highly sensitive to atomistic interface structure, band alignment, and tunneling effects, and they emphasize that standard DFT bandgap errors can significantly affect transport predictions. They further highlight Fermi-level pinning and the limitations of simple band-alignment rules, reinforcing the need for careful interface modeling and beyond-standard-DFT treatments for reliable barrier predictions.

Most recently, Nangoi et al. performed first-principles SBH calculations for Al(111)/Si(111) and CoSi_2_(111)/Si­(111) interfaces using the potential-alignment method.^18^ Using hybrid-functional bulk references, they showed that SBHs can be strongly structure-dependent in systems such as CoSi_2_/Si, due to more covalent interfacial bonding, while remaining relatively insensitive to interface details in Al/Si. This highlights the strong sensitivity of SBH predictions to interface construction, electrostatic potential alignment, and the choice and accuracy of bulk reference calculations.

Therefore, there is a clear need for a systematic and physically grounded evaluation of the computational strategies used to predict Schottky barriers. In this study, we focus on Si/Metal interfaces as a representative test sample to dissect the role of XC functional choice, reference bulk protocols, structural strain, and relativistic effects in Schottky barrier calculations. By benchmarking multiple computational routes against experimental data and analyzing the sources of error, we aim to define a reliable and transferable methodology for Schottky barrier prediction that can serve as a foundation for future large-scale screening and interface design efforts.

The present work addresses ideal, abrupt, and stoichiometric interfaces within a local potential-alignment framework. Accordingly, the reported SBHs should be interpreted as intrinsic, interface-limited quantities, rather than full device-level barriers including long-range depletion electrostatics.

More generally, two levels of Schottky-barrier theory should be distinguished. The first is the local band-alignment or interface-dipole picture adopted here, in which the barrier is defined from the relative alignment of the metal Fermi level, semiconductor band edges, and local electrostatic lineup at an ideal interface. The second is a fully self-consistent Schottky model, in which the barrier height, band bending, and depletion width emerge from the coupled solution of the interfacial electronic structure and macroscopic electrostatics. In this context, Skachkov et al. showed that the Fermi-level position at a metal–semiconductor contact is inseparable from the macroscopic electrostatic potential, and that realistic SB physics requires a self-consistent Poisson-DFT treatment including dopants, free carriers, and metal-induced gap states.^19^

## 2 Methods

### 2.1 Workflow Overview

Figure 1 presents an overview of the computational procedure used to evaluate Schottky barrier heights at metal–semiconductor interfaces. The framework builds upon the Joint Automated Repository for Various Integrated Simulations density functional theory database (**JARVIS-DFT**).^20,21^ The approach consists of four main steps: (i) bulk electronic structure calculations to obtain the VBM of the semiconductor and the E_*F*_ of the metal, (ii) construction of lattice-matched interface models, (iii) calculation of the electrostatic potential profile across the interface, and (iv) extraction of the Schottky barrier height using the potential alignment formalism.

**Figure 1:**
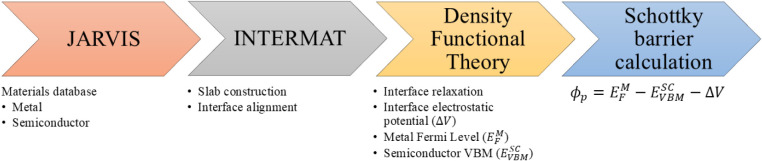
Schematic overview of the workflow. Based on the JARVIS-DFT repository, the VBM and E*_F_* are calculated for semiconductors (SC) and metals (M), respectively. Interfaces are generated from the bulk counterparts using the Zur algorithm and ALIGNN-FF. The workflow aims to automate Schottky barrier height calculations.

### 2.2 Interface Construction

To build the M-SC interfaces, we use a combination of DFT and machine learning (ML). This effort builds upon our previous work, **InterMat**,^22^ a materials interface computational framework designed to predict band offsets of semiconductor interfaces using density functional theory and graph neural networks. In InterMat, we employed a combination of DFT, the **Zur interface generation algorithm**,^23^ and atomistic line graph neural networks (**ALIGNN**)^24^ to predict semiconductor-semiconductor (SC-SC) band offsets using both alternate slab junction (ASJ) and independent unit (IU) models.

This work adapts this framework to M-SC systems by generating interfaces using data from the JARVIS-DFT database, applying the Zur algorithm, and prerelaxing structures with **ALIGNN-FF**, the Atomistic Line Graph Neural Network force field,^25^ enabling efficient screening of large metal–semiconductor combinatorial spaces.

The process begins by selecting surface slabs of metals and semiconductors from the JARVIS-DFT repository and defining the interface parameters. These include maximum lattice mismatch (8%), maximum area (75 Å^2^), angular tolerance (1°), distance between substrate and film, total cell height, and in-plane displacement interval (0.1 Å).

Given the importance of the distance between substrate and film, which controls the interfacial bonding interaction and strongly affects the electrostatic potential profile, we determined the optimal separation for each interface using DFT. Calculations were performed by varying the distance from 1.5 to 3.0 Å in steps of 0.1 Å, selecting the distance that minimized the total energy (see Supporting Information).

The cell height was optimized with respect to the electrostatic potential by monitoring changes in the planar-averaged potential of the silicon and metal regions in the interface. Calculations were performed by increasing the number of Si bilayers in the interface from 3 to 7 bilayers, while keeping the metal counterpart at a comparable thickness. Changes in the electrostatic potential were found to be minimized for 7 Si bilayers, corresponding to a total cell height of approximately 43.0–43.7 Å for both the metal and semiconductor regions.

Candidate interfaces are constructed by stacking the surface slabs along the *z*-direction. To minimize computational cost, a grid search over in-plane displacements is performed using the ALIGNN-FF machine-learned force field, enabling efficient prerelaxation of the structures prior to final optimization with DFT.

### 2.3 Schottky Barrier Formalism

The Schottky barrier height is defined as the energy difference between the metal Fermi level and the band edges of the semiconductor. For an ideal, unpinned interface, the hole and electron barriers are given by

1 $$\Phi_{p}=E_{F}^{M}-E_{\mathrm{VBM}}^{\mathrm{SC}}-\Delta V$$

2 $$\Phi_{n}=E_{g}-\Phi_{p}$$

where E_*F*_ is the metal Fermi level, E_VBM_ is the valence band maximum of the semiconductor, E_*g*_ is the semiconductor bandgap, and ΔV is the electrostatic potential difference across the interface. All quantities are referenced to the average electrostatic potential in their respective bulk regions. The definitions are illustrated schematically in Figure 2.

**Figure 2:**
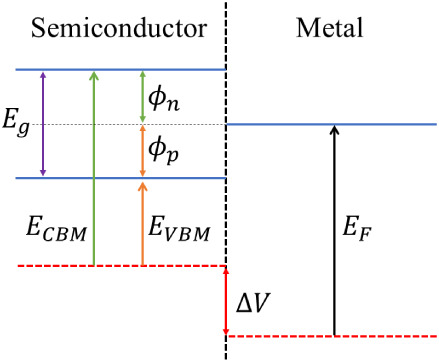
Schematic representation of the Schottky barrier at a metal–semiconductor junction.

The local electrostatic potential V­(x,y,z) is extracted from the DFT-calculated Hartree plus ionic potential. To obtain the macroscopic average V̿(z) used for band alignment, we perform a two-step averaging.

First, the planar average *V̅* (z) of the local potential is computed over the cross-sectional area of the interface *S*:

3 $$\mathrm{V̅}\left(z\right)=\frac{1}{S}\int_{S}V\left(x,\;y,\;z\right)dx\;dy$$

Then, the macroscopic average V̿(*z*) is calculated over a repeat length *L* located in the middle region of the substrate/film:

4 $$\mathrm{V̅̅}\left(z\right)=\frac{1}{L}\int_{-L/2}^{L/2}\mathrm{V̅}\left(z+z^{\prime }\right)dz^{\prime }$$

Finally, the electrostatic potential offset is calculated as

5 $$\Delta V={\mathrm{V̅̅}}_{\mathrm{SC}}-{\mathrm{V̅̅}}_{M}$$

where a positive ΔV indicates that the metal E_*F*_ lies within the semiconductor E_*g*_.

In the present work, the Schottky barrier is defined within a local potential-alignment framework in which the electrostatic contribution is limited to the interface dipole term Δ*V*. Because the semiconductor is represented by a finite slab, the calculations do not include a spatially extended depletion or inversion region, nor a self-consistent solution of Poisson electrostatics coupled to dopants, defects, or free carriers. Accordingly, defect, dopant, and carrier concentrations are not treated as explicit charged degrees of freedom, and the relative alignment of the metal Fermi level and semiconductor band edges should not be interpreted as arising from a self-consistent redistribution of charge in the semiconductor. Rather, the barriers reported here should be interpreted as intrinsic interface-alignment quantities appropriate for ideal, abrupt, stoichiometric interfaces, rather than full device-level Schottky barriers including long-range band bending and carrier-dependent electrostatics.

Figure 3 (top panel) shows the planar-averaged (blue) and macroscopic-averaged (red) electrostatic potential of the Si(111)/Al(111) interface along the *z*-axis. The vertical dashed lines indicate the boundaries of the interfacial region between the Si and Al layers. The periodic oscillations in the potential reflect the atomic layering within each material, as illustrated in the lower panel of Figure 3, which displays the corresponding atomic structure of the interface.

**Figure 3:**
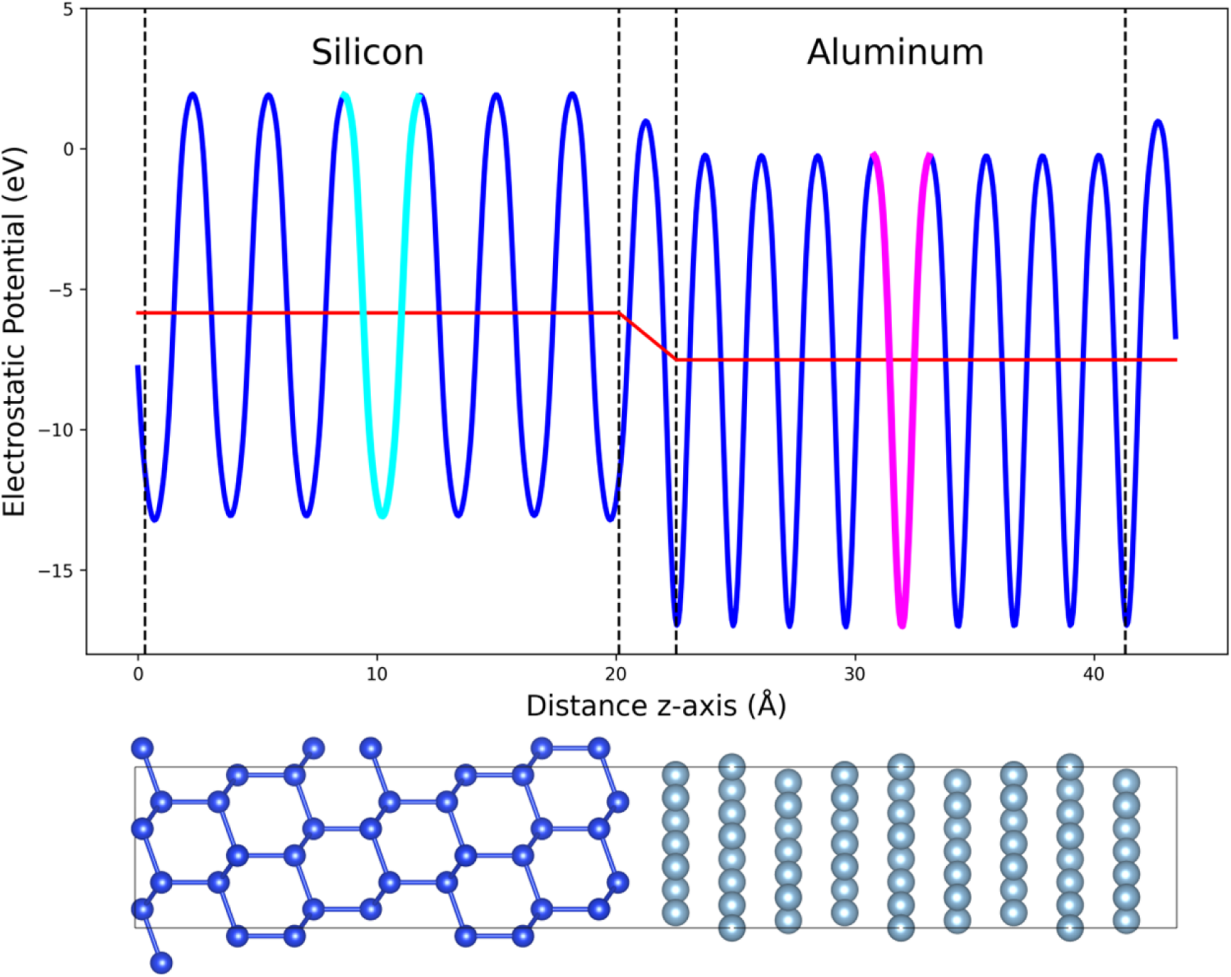
(Top) Planar-averaged (blue) and macroscopically averaged (red) electrostatic potentials of the Si(111)/Al(111) interface. Vertical dashed black lines indicate the positions where the Si and Al layers start and end along the *z*-axis. Cyan and magenta lines denote the repeat unit layers on the left and right sides, respectively. (Bottom) Atomic structure of the Si(111)/Al(111) interface. Si atoms are shown in blue, and Al atoms in gray.^26^.

A key feature of this profile is the electrostatic potential offset across the interface, which corresponds to the dipole term ΔV in the band alignment equation. The macroscopic average enables identification of bulk-like regions on either side of the interface, where the potential becomes approximately flat. This is essential for accurately referencing the band edges of each material. In the figure, the cyan and magenta curves highlight the bulk-like regions of Si and Al, respectively, and serve as the basis for extracting V̿(*z*) in the calculation of ΔV.

To complement the 1D profile, Figure 4 shows a 3D electrostatic isosurface. This visualization captures the spatial variation of electrostatic potential and highlights the interface dipole layer. The isosurface representation is especially useful for detecting localized charge accumulations or discontinuities, which may not be evident in planar-averaged profiles.

**Figure 4:**
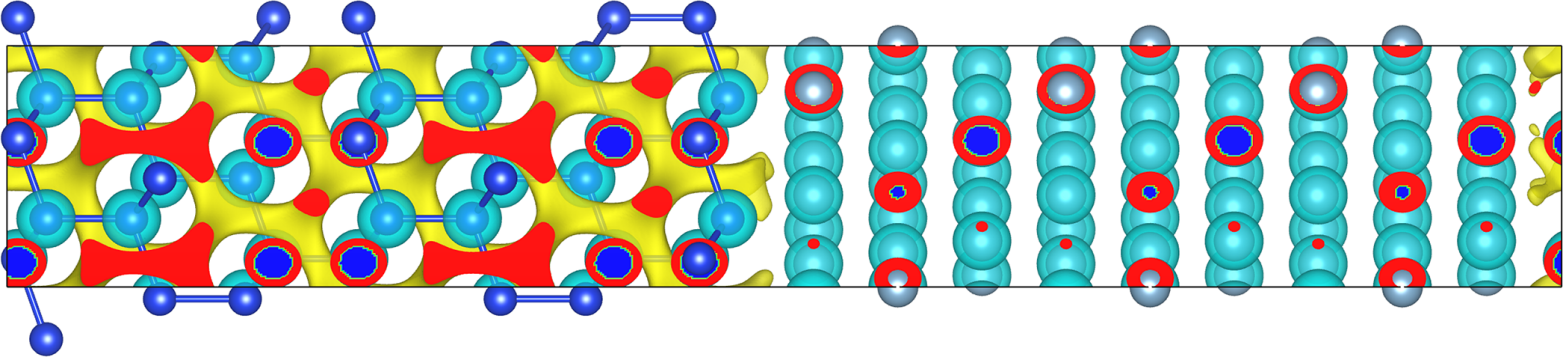
Electrostatic potential isosurface of the Si(111)/Al(111) interface rendered in VESTA (Visualization for Electronic and Structural Analysis) at an isovalue of 9.5 eV.^26^

### 2.4 DFT Details

All density functional theory calculations were performed using the Vienna *Ab Initio* Simulation Package (VASP).^27,28^ The projector augmented-wave (PAW) method was employed together with several XC functionals, including Perdew–Burke–Ernzerhof (PBE),^11^ OptB88vdW (OPT),^29^ Strongly Constrained and Appropriately Normed (SCAN),^30^ modified Becke-Johnson (mBJ),^31^ and Heyd-Scuseria-Ernzerhof (HSE, 2006 version).^32^

All calculations used a plane-wave cutoff of 600 eV and Monkhorst–Pack *k*-point meshes that were each converged to better than 1 meV/atom. For all interface supercells, a 7 × 7 × 1 grid was found sufficient to converge both the total energy and the electrostatic potential profile.

Interface relaxations were done at a fixed volume allowing atoms to move (ISIF = 2), consistent with the InterMat framework in which the semiconductor acts as the substrate, while the metal film accommodates most of the lattice mismatch.

## 3 Results and Discussion

### 3.1 Identification of Stable Interface Configurations

As a first step, we evaluated the structural stability of the generated metal–semiconductor interfaces in order to identify the most favorable atomic configurations for subsequent Schottky barrier calculations. For each metal (M = Al, Cu, Ag, Au), we constructed Si(111)/M(100), Si(111)/M(110), and Si(111)/M(111) interfaces and assessed their stability using both formation energy and work of adhesion criteria.

The formation energy of a M-SC interface is defined as the energy required to form the interface from its constituent relaxed bulk phases,

6 $$E_{\mathrm{formation}}=E_{\mathrm{interface}}-N_{\mathrm{SC}}E_{\mathrm{SC}}^{\mathrm{relaxed}}-N_{M}E_{M}^{\mathrm{relaxed}}$$

where E_interface_ is the total energy of the interface supercell, *E*_SC_^relaxed^ and *E*_M_^relaxed^ are the energies per atom of the relaxed bulk semiconductor and metal, respectively, and *N*_SC_ and *N*_M_ are the corresponding numbers of atoms in the interface cell.

To account for the presence of two interfaces in the periodic supercell and to enable comparison across different interface sizes, we further compute the work of adhesion (*W*_ad_) as^33^

7 $$W_{\mathrm{ad}}=\frac{N_{\mathrm{SC}}E_{\mathrm{SC}}^{\mathrm{relaxed}}+N_{M}E_{M}^{\mathrm{relaxed}}-E_{\mathrm{interface}}}{2A}$$

where *A* is the interfacial area. The work of adhesion is the reversible work required to separate two contacting phases from their equilibrium interfacial distance to infinite separation. Thus, larger W_ad_ values correspond to stronger interfacial bonding and greater interface stability. The calculated W_ad_ includes contributions from interfacial bonding and residual elastic strain associated with lattice matching, and therefore serves as a practical measure of interface stability.

All stability calculations were performed using self-consistent PBE calculations for computational efficiency. For all metals in this study, the Si(111)/M(111) interface was found to be the most stable configuration, exhibiting the lowest formation energy and highest work of adhesion compared to the (100) and (110) terminations. Detailed numerical values for all configurations are reported in Table S1 of the Supporting Information.

Based on these results, all subsequent electronic structure and Schottky barrier calculations in this work focus exclusively on the Si(111)/M(111) interfaces.

### 3.2 Schottky Barrier Calculations

Figure 5(a) shows the layer-resolved local density of states (LDOS) of the Al/Si interface computed with PBE. The low-LDOS (white) region in the middle of the Si slab corresponds to the Si bandgap. However, a strictly vanishing LDOS is not recovered. As seen in Figure 5(b–e), the Si layers progressively develop a gap-like feature around the Fermi level, but residual spectral weight persists in the nominal gap. This behavior is mainly attributed to metal-induced gap states (MIGS) and Al–Si wave function hybridization at the interface, and it can be enhanced by the finite smearing used in the DOS calculation. In the present framework, however, MIGS are analyzed only through their local spectral signature in the interface LDOS and are not separated as a spatially decaying charge contribution to long-range band bending in the semiconductor. Notably, the gap-like Si region improves from PBE to OPT to SCAN. In contrast, mBJ deviates from this trend, showing an excessive upward shift of the Fermi level that yields an artificially metallic LDOS in the middle of the Si slab.

**Figure 5:**
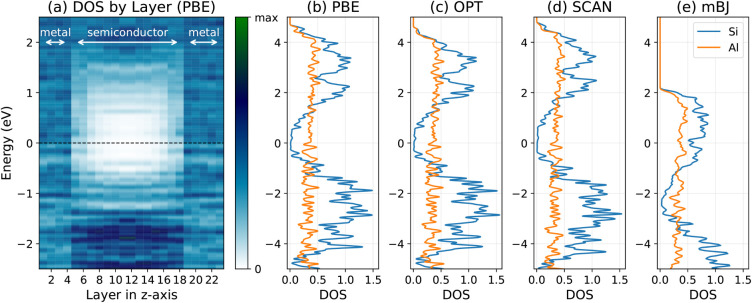
Local density of states (LDOS) of the Al/Si interface computed with different XC functionals: (a) layer-resolved LDOS along the interface normal (*z*-axis) using PBE; (b–e) LDOS for the central layers of the Al and Si slabs computed with (b) PBE, (c) OPT, (d) SCAN, and (e) mBJ. In all panels, energies are referenced to the Fermi level (*E_F_* = 0).

As shown in Table 1, we evaluate the performance of several XC approaches for predicting SBHs at Si/Metal interfaces, including semilocal (PBE, OPT), meta-GGA (SCAN), modified Becke-Johnson (mBJ), and mixed hybrid-semilocal schemes (HSE+PBE, HSE+OPT, HSE+SCAN). For the mBJ calculations, we used a fixed setting of β = 0.9797 with the default α parameter for the interface, bulk-Si, and bulk-metal calculations. The β value was chosen to reproduce the experimental Si bandgap (*E*_*g*_ = 1.17 eV), and the mBJ calculations were performed on structures taken from previously relaxed OPT calculations. The mixed hybrid-semilocal schemes denote calculations in which the interface electrostatic lineup ΔV is obtained from a semilocal XC functional (PBE, OPT, or SCAN), while the bulk reference quantities are computed using HSE. In these HSE calculations, the Si exact-exchange fraction was set to α = 0.23 to reproduce E_g_ = 1.17 eV, whereas the metals were evaluated with α = 0 to avoid introducing artificial exchange effects. No explicit HSE interface calculations were performed due to their excessive computational cost. In addition, we assess three distinct reference protocols for extracting bulk quantities entering the potential-alignment formalism: (A) relaxed bulk silicon and metal calculations, (B) relaxed bulk silicon and metal calculations including spin–orbit coupling (SOC), and (C) strained, vacuum-free bulk silicon and metal calculations consistent with the interface geometry.

**Table 1:** Schottky Barrier Heights (eV) for Si(111)/M(111) Interfaces Computed Using Different XC Functionals and Reference Protocols^a^

Metal	PBE	OPT	SCAN	mBJ	HSE+PBE	HSE+OPT	HSE+SCAN	Expt
Procedure A (relaxed)								
Al	0.58	0.71	0.88	0.56	0.75	0.94	0.91	0.58
Cu	–0.27	–0.09	0.15	0.71	–0.10	0.13	–0.01	0.46
Ag	0.01	–0.08	0.17	1.10	0.18	–0.05	–0.10	0.55
Au	–0.12	–0.12	0.11	0.76	0.05	0.10	–0.15	0.34
MAE	0.43	0.44	**0.31**	**0.31**	0.35	0.38	0.49	–
Procedure B (relaxed+SOC)								
Al	0.56	0.69	0.86	0.55	0.74	0.94	0.91	0.58
Cu	–0.29	–0.10	0.16	0.70	–0.14	0.08	–0.05	0.46
Ag	0.01	–0.08	0.17	1.09	0.18	–0.06	–0.10	0.55
Au	0.04	0.03	0.26	0.76	0.20	0.25	–0.01	0.34
MAE	0.40	0.40	**0.26**	0.31	0.32	0.36	0.46	–
Procedure C (strained)								
Al	0.00	0.28	0.36	0.12	0.36	0.60	0.57	0.58
Cu	0.03	0.19	–0.01	0.92	0.39	0.61	0.46	0.46
Ag	0.20	–0.06	–0.04	0.73	0.56	0.33	0.28	0.55
Au	0.13	0.18	–0.22	1.05	0.49	0.54	0.28	0.34
MAE	0.39	0.34	0.46	0.45	0.11	0.15	**0.09**	–

a Procedure A: relaxed bulk references; Procedure B: relaxed bulk references with SOC; Procedure C: strained, vacuum-free bulk references consistent with the interface geometry. Experimental values extracted from ref 34 are shown in the last column. Mean absolute error (MAE) is reported for each XC functional vs experiment. The best MAE in each procedure is bolded.

The experimental values from ref 34 correspond to p-type Si(111) samples with reported bulk doping concentrations in the range 10^14^–10^18^cm^–3^, with barrier heights extracted from both *I*-*V* and 1/*C* ^2^-*V* measurements. These values are included as literature benchmarks and should not be interpreted as implying a strict one-to-one correspondence with the intrinsic interface-limited barriers computed here.

### 3.3 Relaxed Bulk Reference Calculations

Using relaxed bulk references **(Procedure A)**, PBE and OPT systematically underestimate SBHs and frequently predict negative barriers for Cu-, Ag-, and Au-based interfaces, indicating unphysical metallic alignment. SCAN and mBJ yield uniformly positive SBHs, but exhibit distinct biases: SCAN tends to underestimate barriers, while mBJ significantly overestimates SBHs for noble metals due to an unphysical upward shift of the metal E_*F*_. Overall, SCAN provides the most balanced performance within Procedure A, albeit with moderate mean absolute errors.

### 3.4 Including Spin–Orbit Coupling in Bulk References

Including SOC in relaxed bulk reference calculations **(Procedure B)** primarily affects heavy metals, most notably Au. While SOC improves the SBH prediction for Au in selected approaches, it does not systematically resolve negative barriers or alignment inconsistencies across all methods. In particular, PBE, OPT, and mixed approaches that already suffer from unphysical SBHs remain problematic. SCAN with SOC shows a modest reduction in error relative to Procedure A, whereas mBJ retains its overestimation trend. These results indicate that SOC provides a secondary correction that is insufficient on its own to ensure reliable SBH predictions.

### 3.5 Strained, Vacuum-Free Bulk References

The most significant improvement in both physical consistency and quantitative accuracy is achieved using strained, vacuum-free bulk silicon and metal calculations consistent with the interface geometry **(Procedure C)**. Under this protocol, several approaches that previously produced negative or scattered SBHs yield uniformly positive and substantially more accurate results. This highlights the critical importance of using reference bulk calculations that preserve the strain state and periodicity imposed by the interface.

Within Procedure C, mixed hybrid-semilocal approaches clearly outperform purely semilocal or meta-GGA functionals. In particular, HSE+SCAN provides the lowest overall error, closely reproducing experimental SBHs across all interfaces. HSE+PBE and HSE+OPT achieve comparable accuracy with slightly higher errors, but at reduced computational cost. In contrast, PBE and mBJ, while yielding positive barriers under this protocol, remain less accurate overall.

Taken together, these results demonstrate that structural and electrostatic consistency between interface and bulk reference calculations is the dominant factor governing SBH accuracy, outweighing the isolated impact of SOC or XC functional choice alone. While HSE+SCAN combined with Procedure C provides the highest accuracy, the computational cost of SCAN-based interface calculations limits its scalability. As a practical compromise for high-throughput screening, we identify HSE+PBE with strained, vacuum-free bulk references (Procedure C) as an optimal choice, delivering near-benchmark accuracy with substantially reduced cost.

SOC may be applied selectively for heavy-metal contacts such as Au, where relativistic effects are expected to be significant, but is not required for the default high-throughput workflow.

## 4 Conclusion

In this work, we systematically assessed the reliability of density functional theory-based approaches for predicting SBHs at Si/Metal interfaces using the potential alignment method. By benchmarking multiple XC functionals and reference protocols against experimental data for Si(111)/Metal (Al, Cu, Ag, and Au) interfaces, we identified the key factors governing both physical consistency and quantitative accuracy.

We show that commonly used semilocal and nonlocal functionals such as PBE and OPT frequently yield unphysical (negative) SBHs, rendering them unsuitable for predictive screening. Meta-GGA SCAN and mBJ consistently produce positive barriers, but with distinct and systematic biases: SCAN provides balanced but underestimated SBHs, while mBJ strongly overestimates barriers for metals due to an artificial upward shift of the metal Fermi level. Inclusion of spin–orbit coupling improves agreement for heavy metals such as Au, but does not resolve fundamental alignment errors and is therefore a secondary correction.

The most significant improvement arises from enforcing structural and electrostatic consistency between interface and bulk reference calculations. When bulk silicon and metal references are computed under the same strain conditions as the interface, mixed hybrid-semilocal approaches yield uniformly positive SBHs with substantially reduced error. Among these, HSE+SCAN achieves the highest accuracy, while HSE+PBE provides comparable performance at significantly lower computational cost, making it the preferred choice for high-throughput applications.

Overall, our results demonstrate that accurate Schottky barrier prediction depends more critically on consistent reference protocols than on increasing XC sophistication alone. The proposed framework offers a robust and transferable strategy for large-scale screening of metal–semiconductor interfaces and can be readily extended to other semiconductors, metals, and interface orientations.

The present results should, however, be interpreted within the scope of a local potential-alignment model. The calculated SBHs describe intrinsic, interface-limited barrier heights for ideal, abrupt, stoichiometric interfaces and do not include spatially extended space-charge regions, explicit dependence on doping or temperature, or a self-consistent treatment of Poisson electrostatics coupled to dopants, defects, free carriers, and spatially decaying MIGS. Accordingly, the reported barriers should not be interpreted as full device-level Schottky barriers.

Within these limitations, this work lays the foundation for data-driven discovery and optimization of contact materials in next-generation electronic and optoelectronic devices. Future extensions of this framework could include coupling the present interface calculations to a Poisson solver to capture macroscopic Schottky potentials, incorporating spatially resolved MIGS contributions to band bending, and embedding the interface region within a multiscale treatment of realistic contacts.

## Supplementary Material



## Data Availability

Structures and related resources supporting this work are available at: https://github.com/vdovale29/Schottky-Barriers.git and https://github.com/usnistgov/chips-Schottky-Barriers.git.

## Notes

Optimal distance between substrate and film; stability of different configurations; details of Schottky barrier calculations (PDF)

## References

[1] Robertson J. (2013). Band offsets,
Schottky barrier heights, and their effects on electronic devices. J. Vacuum Sci. Technol. A.

[2] Milnes, A. G. Heterojunctions and metal semiconductor junctions; Elsevier, 2012.

[3] Schottky W. (1926). Small-shot
effect and flicker effect. Phys. Rev..

[4] Bardeen J. (1947). Surface states
and rectification at a metal semiconductor contact. Phys. Rev..

[5] Tung R. T. (2014). The physics
and chemistry of the Schottky barrier height. Appl. Phys. Rev..

[6] Choudhary K. (2025). Slakonet:
A unified Slater-Koster tight-binding framework using neural network
infrastructure for the periodic table. J. Phys.
Chem. Lett..

[7] Kohn W., Sham L. J. (1965). Self-consistent equations including
exchange and correlation
effects. Phys. Rev..

[8] Dirac, P. A. Note on exchange phenomena in the Thomas atom. In Mathematical proceedings of the Cambridge philosophical society; Cambridge University Press, 1930, Vol. 26, pp. 376–385.

[9] Ceperley D. M., Alder B. J. (1980). Ground state of the electron gas
by a stochastic method. Phys. Rev. Lett..

[10] Perdew J. P., Zunger A. (1981). Self-interaction correction
to density-functional approximations
for many-electron systems. Phys. Rev. B.

[11] Perdew J. P., Burke K., Ernzerhof M. (1996). Generalized
gradient approximation
made simple. Phys. Rev. Lett..

[12] Weston L., Tailor H., Krishnaswamy K., Bjaalie L., Van de Walle C. G. (2018). Accurate
and efficient band-offset calculations from density functional theory. Comput. Mater. Sci..

[13] Hinuma Y., Grüneis A., Kresse G., Oba F. (2014). Band alignment of semiconductors
from density-functional theory and many-body perturbation theory. Phys. Rev. B.

[14] Delaney K. T., Spaldin N. A., Van de Walle C. G. (2010). Theoretical
study of Schottky-barrier
formation at epitaxial rare-earth-metal/semiconductor interfaces. Phys. Rev. B.

[15] Stengel M., Aguado-Puente P., Spaldin N. A., Junquera J. (2011). Band alignment
at metal/ferroelectric
interfaces: Insights and artifacts from first principles. Phys. Rev. B.

[16] Wang X., Zebarjadi M., Esfarjani K. (2016). First principles
calculations of
solid-state thermionic transport in layered van der Waals heterostructures. Nanoscale.

[17] Wang X., Zebarjadi M., Esfarjani K. (2018). High-performance solid-state thermionic
energy conversion based on 2D van der Waals heterostructures: A firstprinciples
study. Sci. Rep..

[18] Nangoi J. K., Palmstrøm C. J., Van de Walle C. G. (2024). First-principles
studies of Schottky
barriers and tunneling properties at Al(111)/Si(111) and CoSi2(111)/Si(111)
interfaces. Phys. Rev. B.

[19] Skachkov D., Liu S. L., Wang Y., Zhang X. G., Cheng H. P. (2021). First-principles
theory for Schottky barrier physics. Phys. Rev.
B.

[20] Choudhary K., Garrity K. F., Reid A. C., DeCost B., Biacchi A. J., Hight Walker A. R., Trautt Z., Hattrick-Simpers J., Kusne A. G., Centrone A. (2020). The joint automated
repository for various integrated simulations (JARVIS) for data-driven
materials design. Npj Comput. Mater..

[21] Choudhary K. (2025). The JARVIS
infrastructure is all you need for materials design. Comput. Mater. Sci..

[22] Choudhary K., Garrity K. F. (2024). InterMat: accelerating band offset prediction in semiconductor
interfaces with dft and deep learning. Digital
Discovery.

[23] Zur A., McGill T. C. (1984). Lattice match: An application to heteroepitaxy. J. Appl. Phys..

[24] Choudhary K., DeCost B. (2021). Atomistic line graph
neural network for improved materials
property predictions. Npj Comput. Mater..

[25] Choudhary K., DeCost B., Major L., Butler K., Thiyagalingam J., Tavazza F. (2023). Unified graph neural
network force-field for the periodic
table: solid state applications. Digital Discovery.

[26] Momma K., Izumi F. (2011). VESTA3 for three-dimensional
visualization of crystal, volumetric
and morphology data. J. Appl. Crystallogr..

[27] Kresse G., Furthmüller J. (1996). Efficient iterative schemes for ab initio totalenergy
calculations using a plane-wave basis set. Phys.
Rev. B.

[28] Kresse G., Furthmüller J. (1996). Efficiency of ab-initio total energy calculations for
metals and semiconductors using a plane-wave basis set. Comput. Mater. Sci..

[29] Klimeš J., Bowler D. R., Michaelides A. (2010). Chemical accuracy
for the van der
Waals density functional. J. Phys.: Condens.
Matter.

[30] Sun J., Ruzsinszky A., Perdew J. P. (2015). Strongly constrained and appropriately
normed semilocal density functional. Phys. Rev.
Lett..

[31] Tran F., Blaha P. (2009). Accurate band gaps of semiconductors and insulators with a semilocal
exchange-correlation potential. Phys. Rev. Lett..

[32] Krukau A. V., Vydrov O. A., Izmaylov A. F., Scuseria G. E. (2006). Influence of the
exchange screening parameter on the performance of screened hybrid
functionals. J. Chem. Phys..

[33] Ebnesajjad, S. ; Ebnesajjad, C. Surface treatment of materials for adhesive bonding; William Andrew, 2013.

[34] Smith B. L., Rhoderick E. H. (1971). Schottky barriers on p-type silicon. Solid-State Electron..

