# Supplementary material for: Effect of Exchange-Correlation Functionals on Schottky Barriers at Si/Metal Interfaces
Source: J Phys Chem C Nanomater Interfaces. 2026 Jun 3;130(24):8539–46. doi: 10.1021/acs.jpcc.6c01668 (PMC13288670; doi:10.1021/acs.jpcc.6c01668)
Supplement: Supplementary file 1 [file jp6c01668_si_001.pdf]

Supporting Information

Effect of Exchange-Correlation Functionals on Schottky Barriers at Si/Metal Interfaces

Viviana Dovale-Farelo[1,2] and Kamal Choudhary[1,3,4]

[1]National Institute of Standards and Technology, Gaithersburg, MD, 20899
[2]University of Maryland, College Park, MD, 20742
[3]Department of Materials Science and Engineering, Johns Hopkins University, Baltimore, MD 21218, USA
[4]Department of Electrical and Computer Engineering, Johns Hopkins University, Baltimore, MD 21218, USA

March 2026

1 Optimal Distance Between Substrate and Film

The optimal separation between the substrate (silicon) and film (metal) was determined using DFT. Si(111)/metal interfaces were constructed using different surface orientations for the metallic component. Calculations were performed with PBE by varying the interfacial distance from 1.5 to 3.0 Å in steps of 0.1 Å. The distance corresponding to the minimum total energy was then selected.

Table S1: Optimal separation distance (Å) between Si(111) and different metal surfaces.

Interface	Metal(100)	Metal(110)	Metal(111)
Si/Al	2.4	2.4	2.4
Si/Cu	2.1	2.0	2.2
Si/Ag	2.5	2.3	2.4
Si/Au	2.5	2.3	2.3

2 Stability of Different Configurations

Using the optimal separation between the substrate and film, the structural stability of the generated metal-semiconductor interfaces was evaluated to identify the most favorable atomic configurations. For each metal ((M=) Al, Cu, Ag, Au), Si(111)/M(100), Si(111)/M(110), and Si(111)/M(111) interfaces were constructed, and their stability was assessed using both formation energy and work of adhesion criteria.

Table S2: Formation energy (E_{for}, in eV) and work of adhesion (W_{ad}, in eV/Å^2) for Si(111)/Metal interfaces. The table reports the interface system, total interface energy ($E_{interface}$, in eV), relaxed bulk energies of Si ($E_{SC}^{relaxed}$) and the metal ($E_M^{relaxed}$), number of Si (N_{SC}) and metal (N_M) atoms in the interface, and the interfacial area (in Å^2).

**System**	$E_{interface}$	N_{SC}	N_M	$E_{SC}^{relaxed}$	$E_M^{relaxed}$	Area	E_{for}	W_{ad}
Si(111)/Al(111)	-534.47	56	63	-5.43	-3.75	52.27	5.28	-0.05
Si(111)/Al(100)	-138.66	14	20	-5.43	-3.75	13.07	12.20	-0.47
Si(111)/Al(110)	-738.59	84	80	-5.43	-3.75	78.41	16.73	-0.11
Si(111)/Cu(111)	-487.29	42	70	-5.43	-3.75	39.20	2.96	-0.04
Si(111)/Cu(100)	-298.79	28	40	-5.43	-3.75	26.14	3.05	-0.06
Si(111)/Cu(110)	-744.81	84	80	-5.43	-3.75	78.41	10.78	-0.07
Si(111)/Ag(111)	-471.34	56	63	-5.43	-2.72	52.27	3.79	-0.04
Si(111)/Ag(100)	-145.99	28	40	-5.43	-2.72	26.14	114.69	-2.19
Si(111)/Ag(110)	-652.13	84	80	-5.43	-2.72	78.41	21.13	-0.13
Si(111)/Au(111)	-504.78	56	63	-5.43	-3.22	52.27	1.96	-0.02
Si(111)/Au(100)	-1.91	14	20	-5.43	-3.22	13.07	138.47	-5.30
Si(111)/Au(110)	-688.48	84	80	-5.43	-3.22	78.41	24.92	-0.16

3 Details of the Schottky Barrier Calculations

The following table summarizes the Schottky barrier (Φ_p, in eV) calculations for Si(111)/M(111) interfaces obtained using different exchange-correlation functionals (XC). The Schottky barrier was calculated as

$$\Phi_p = E_F^M - E_{\mathrm{VBM}}^{\mathrm{SC}} - \Delta V. \tag{1}$$

The semiconductor (SC) and metal (M) bulk reference types used in the calculations are specified as follows:

- Relaxed: semiconductor and metal bulk references were relaxed to their equilibrium positions.
- SOC: semiconductor and metal bulk references were relaxed to their equilibrium positions including spin-orbit coupling.
- Strained: semiconductor and metal bulk references were strained, vacuum-free, and consistent with the interface geometry.

The metal Fermi energy (E_F^M) and the semiconductor valence band maximum ($E_{\mathrm{VBM}}^{\mathrm{SC}}$) are provided in eV.

The electrostatic potentials of the semiconductor and metal in the interface, V_{SC} and V_{M}, respectively, together with the electrostatic potential difference (ΔV), are reported in eV. The calculated Schottky barrier values are compared with experimental results [1] using the mean absolute error (MAE).

Table S3:

XC	SC	M	Type	V_{SC}	V_M	ΔV	E_F^M	E_{VBM}^{SC}	Φ_p	Expt.	MAE
PBE	Si	Al	Relaxed	-5.92	-7.80	1.88	8.07	5.62	0.58	0.58	0.00
OPT	Si	Al	Relaxed	-5.88	-7.56	1.68	7.91	5.52	0.71	0.58	0.13
SCAN	Si	Al	Relaxed	-5.90	-7.61	1.71	8.27	5.68	0.88	0.58	0.30
mBJ	Si	Al	Relaxed	-5.55	-7.52	1.97	7.69	5.16	0.56	0.58	0.02
PBE	Si	Cu	Relaxed	-5.67	-7.15	1.47	6.82	5.62	-0.27	0.46	0.73
OPT	Si	Cu	Relaxed	-5.75	-7.00	1.25	6.68	5.52	-0.09	0.46	0.55
SCAN	Si	Cu	Relaxed	-5.54	-6.92	1.39	7.22	5.68	0.15	0.46	0.31
mBJ	Si	Cu	Relaxed	-5.68	-7.75	2.07	7.94	5.16	0.71	0.46	0.25
PBE	Si	Ag	Relaxed	-5.72	-7.93	2.21	7.84	5.62	0.01	0.55	0.54
OPT	Si	Ag	Relaxed	-5.63	-8.08	2.45	7.89	5.52	-0.08	0.55	0.63
SCAN	Si	Ag	Relaxed	-5.60	-8.09	2.49	8.35	5.68	0.17	0.55	0.38
mBJ	Si	Ag	Relaxed	-5.64	-8.72	3.08	9.34	5.16	1.10	0.55	0.55
PBE	Si	Au	Relaxed	-5.73	-8.82	3.08	8.58	5.62	-0.12	0.34	0.46
OPT	Si	Au	Relaxed	-5.68	-8.72	3.03	8.44	5.52	-0.12	0.34	0.46
SCAN	Si	Au	Relaxed	-5.60	-8.89	3.29	9.09	5.68	0.11	0.34	0.23
mBJ	Si	Au	Relaxed	-5.78	-9.10	3.32	9.23	5.16	0.76	0.34	0.42
HSE+PBE	Si	Al	Relaxed	-5.92	-7.80	1.88	8.07	5.45	0.75	0.58	0.17

Table S3:

XC	SC	M	Type	V_{SC}	V_M	ΔV	E_F^M	E_{VBM}^{SC}	Φ_p	Expt.	MAE
HSE+OPT	Si	Al	Relaxed	-5.88	-7.56	1.68	8.07	5.45	0.94	0.58	0.36
HSE+SCAN	Si	Al	Relaxed	-5.90	-7.61	1.71	8.07	5.45	0.91	0.58	0.33
HSE+PBE	Si	Cu	Relaxed	-5.67	-7.15	1.47	6.82	5.45	-0.10	0.46	0.56
HSE+OPT	Si	Cu	Relaxed	-5.75	-7.00	1.25	6.82	5.45	0.13	0.46	0.33
HSE+SCAN	Si	Cu	Relaxed	-5.54	-6.92	1.39	6.82	5.45	-0.01	0.46	0.47
HSE+PBE	Si	Ag	Relaxed	-5.72	-7.93	2.21	7.84	5.45	0.18	0.55	0.37
HSE+OPT	Si	Ag	Relaxed	-5.63	-8.08	2.45	7.84	5.45	-0.05	0.55	0.60
HSE+SCAN	Si	Ag	Relaxed	-5.60	-8.09	2.49	7.84	5.45	-0.10	0.55	0.65
HSE+PBE	Si	Au	Relaxed	-5.73	-8.82	3.08	8.59	5.45	0.05	0.34	0.29
HSE+OPT	Si	Au	Relaxed	-5.68	-8.72	3.03	8.59	5.45	0.10	0.34	0.24
HSE+SCAN	Si	Au	Relaxed	-5.60	-8.89	3.29	8.59	5.45	-0.15	0.34	0.49
PBE	Si	Al	SOC	-5.92	-7.80	1.88	8.07	5.63	0.56	0.58	0.02
OPT	Si	Al	SOC	-5.88	-7.56	1.68	7.91	5.54	0.69	0.58	0.11
SCAN	Si	Al	SOC	-5.90	-7.61	1.71	8.27	5.70	0.86	0.58	0.28
mBJ	Si	Al	SOC	-5.55	-7.52	1.97	7.69	5.17	0.55	0.58	0.03
PBE	Si	Cu	SOC	-5.67	-7.15	1.47	6.81	5.63	-0.29	0.46	0.75
OPT	Si	Cu	SOC	-5.75	-7.00	1.25	6.68	5.54	-0.10	0.46	0.56
SCAN	Si	Cu	SOC	-5.54	-6.92	1.39	7.25	5.70	0.16	0.46	0.30
mBJ	Si	Cu	SOC	-5.68	-7.75	2.07	7.94	5.17	0.70	0.46	0.24
PBE	Si	Ag	SOC	-5.72	-7.93	2.21	7.85	5.63	0.01	0.55	0.54
OPT	Si	Ag	SOC	-5.63	-8.08	2.45	7.90	5.54	-0.08	0.55	0.63
SCAN	Si	Ag	SOC	-5.60	-8.09	2.49	8.36	5.70	0.17	0.55	0.38
mBJ	Si	Ag	SOC	-5.64	-8.72	3.08	9.33	5.17	1.09	0.55	0.54
PBE	Si	Au	SOC	-5.73	-8.82	3.08	8.75	5.63	0.04	0.34	0.30
OPT	Si	Au	SOC	-5.68	-8.72	3.03	8.60	5.54	0.03	0.34	0.31
SCAN	Si	Au	SOC	-5.60	-8.89	3.29	9.26	5.70	0.26	0.34	0.08
mBJ	Si	Au	SOC	-5.78	-9.10	3.32	9.25	5.17	0.76	0.34	0.42
HSE+PBE	Si	Al	SOC	-5.92	-7.80	1.88	8.08	5.47	0.74	0.58	0.16
HSE+OPT	Si	Al	SOC	-5.88	-7.56	1.68	8.08	5.47	0.94	0.58	0.36
HSE+SCAN	Si	Al	SOC	-5.90	-7.61	1.71	8.08	5.47	0.91	0.58	0.33
HSE+PBE	Si	Cu	SOC	-5.67	-7.15	1.47	6.80	5.47	-0.14	0.46	0.60
HSE+OPT	Si	Cu	SOC	-5.75	-7.00	1.25	6.80	5.47	0.08	0.46	0.38
HSE+SCAN	Si	Cu	SOC	-5.54	-6.92	1.39	6.80	5.47	-0.05	0.46	0.51
HSE+PBE	Si	Ag	SOC	-5.72	-7.93	2.21	7.86	5.47	0.18	0.55	0.37
HSE+OPT	Si	Ag	SOC	-5.63	-8.08	2.45	7.86	5.47	-0.06	0.55	0.61
HSE+SCAN	Si	Ag	SOC	-5.60	-8.09	2.49	7.86	5.47	-0.10	0.55	0.65
HSE+PBE	Si	Au	SOC	-5.73	-8.82	3.08	8.75	5.47	0.20	0.34	0.14
HSE+OPT	Si	Au	SOC	-5.68	-8.72	3.03	8.75	5.47	0.25	0.34	0.09
HSE+SCAN	Si	Au	SOC	-5.60	-8.89	3.29	8.75	5.47	-0.01	0.34	0.35
PBE	Si	Al	Strained	-5.92	-7.80	1.88	7.36	5.48	0.00	0.58	0.58
OPT	Si	Al	Strained	-5.88	-7.56	1.68	7.35	5.39	0.28	0.58	0.30
SCAN	Si	Al	Strained	-5.90	-7.61	1.71	7.39	5.32	0.36	0.58	0.22

Table S3:

XC	SC	M	Type	V_{SC}	V_M	ΔV	E_F^M	E_{VBM}^{SC}	Φ_p	Expt.	MAE
mBJ	Si	Al	Strained	-5.55	-7.52	1.97	7.12	5.02	0.12	0.58	0.46
PBE	Si	Cu	Strained	-5.67	-7.15	1.47	6.98	5.48	0.03	0.46	0.43
OPT	Si	Cu	Strained	-5.75	-7.00	1.25	6.83	5.39	0.19	0.46	0.27
SCAN	Si	Cu	Strained	-5.54	-6.92	1.39	6.70	5.32	-0.01	0.46	0.47
mBJ	Si	Cu	Strained	-5.68	-7.75	2.07	8.01	5.02	0.92	0.46	0.46
PBE	Si	Ag	Strained	-5.72	-7.93	2.21	7.89	5.48	0.20	0.55	0.35
OPT	Si	Ag	Strained	-5.63	-8.08	2.45	7.77	5.39	-0.06	0.55	0.61
SCAN	Si	Ag	Strained	-5.60	-8.09	2.49	7.77	5.32	-0.04	0.55	0.59
mBJ	Si	Ag	Strained	-5.64	-8.72	3.08	8.83	5.02	0.73	0.55	0.18
PBE	Si	Au	Strained	-5.73	-8.82	3.08	8.70	5.48	0.13	0.34	0.21
OPT	Si	Au	Strained	-5.68	-8.72	3.03	8.60	5.39	0.18	0.34	0.16
SCAN	Si	Au	Strained	-5.60	-8.89	3.29	8.39	5.32	-0.22	0.34	0.56
mBJ	Si	Au	Strained	-5.78	-9.10	3.32	9.39	5.02	1.05	0.34	0.71
HSE+PBE	Si	Al	Strained	-5.92	-7.80	1.88	7.36	5.12	0.36	0.58	0.22
HSE+OPT	Si	Al	Strained	-5.88	-7.56	1.68	7.40	5.12	0.60	0.58	0.02
HSE+SCAN	Si	Al	Strained	-5.90	-7.61	1.71	7.40	5.12	0.57	0.58	0.01
HSE+PBE	Si	Cu	Strained	-5.67	-7.15	1.47	6.98	5.12	0.39	0.46	0.07
HSE+OPT	Si	Cu	Strained	-5.75	-7.00	1.25	6.98	5.12	0.61	0.46	0.15
HSE+SCAN	Si	Cu	Strained	-5.54	-6.92	1.39	6.97	5.12	0.46	0.46	0.00
HSE+PBE	Si	Ag	Strained	-5.72	-7.93	2.21	7.89	5.12	0.56	0.55	0.01
HSE+OPT	Si	Ag	Strained	-5.63	-8.08	2.45	7.89	5.12	0.33	0.55	0.22
HSE+SCAN	Si	Ag	Strained	-5.60	-8.09	2.49	7.89	5.12	0.28	0.55	0.27
HSE+PBE	Si	Au	Strained	-5.73	-8.82	3.08	8.70	5.12	0.49	0.34	0.15
HSE+OPT	Si	Au	Strained	-5.68	-8.72	3.03	8.70	5.12	0.54	0.34	0.20
HSE+SCAN	Si	Au	Strained	-5.60	-8.89	3.29	8.69	5.12	0.28	0.34	0.06

References

[1] BL Smith and EH Rhoderick. Schottky barriers on p-type silicon. *Solid-State Electronics*, 14(1):71–75, 1971.
